# Pretreatment out-of-pocket costs for people with drug-resistant tuberculosis in Bandung, Indonesia

**DOI:** 10.1371/journal.pone.0352658

**Published:** 2026-07-01

**Authors:** Bony Wiem Lestari, Silvi Indriani, Adriana Viola Miranda, Dyah Ayu Nur Safira, Nur Ayu Fitriani, Raden Desy Nurhayati, Mirza Purwitasari, Almira Alifia, Alamanda Larasmanah, Iceu Dimas Kulsum, Arto Yuwono Soeroto

**Affiliations:** 1 Department of Public Health, Faculty of Medicine, Universitas Padjadjaran, Bandung, West Java, Indonesia; 2 Tuberculosis Working Group, Research Center for Care and Control of Infectious Diseases, Universitas Padjadjaran, Bandung, Indonesia; 3 Department of Internal Medicine, Dr. H. A. Rotinsulu Pulmonary Hospital, Bandung, West Java, Indonesia; 4 Community Lung Health Center, Bandung, West Java, Indonesia; 5 Department of Internal Medicine, Faculty of Medicine Universitas Padjadjaran, Dr. Hasan Sadikin General Hospital, Bandung, West Java, Indonesia; 6 Department of Internal Medicine, Division of Respirology and Critical Care Medicine, Faculty of Medicine Universitas Padjadjaran, Dr. Hasan Sadikin General Hospital, Bandung, West Java, Indonesia; Arsi University College of Health Sciences, ETHIOPIA

## Abstract

**Background:**

People with drug-resistant tuberculosis (DR-TB) experience multiple visits to healthcare providers before DR-TB diagnosis and treatment. Our study aimed to quantify pretreatment direct costs and factors associated with higher costs among people with DR-TB.

**Methods:**

Our cross-sectional study aimed to recruit 300 adults with pulmonary DR-TB from three DR-TB referral centers in Bandung, Indonesia, between February 2023 and February 2024. Participants were interviewed using a structured questionnaire regarding their demographic characteristics and out-of-pocket costs for the following categories: administration, chest radiography, laboratory tests, medication, travel, food, and other non-medical costs. Pretreatment out-of-pocket costs were analyzed descriptively, and factors influencing higher costs were examined using quantile regression. Costs were presented in U.S. dollars (USD, $) and reported as medians and interquartile ranges (IQRs).

**Results:**

Among 258 eligible participants, 57.4% were male; median age was 38 years (IQR 27–47.7). A higher proportion of patients resided in rural areas (68.6%) and had initial visits to community health center (CHC) for TB-related symptoms (53.1%). The median pretreatment direct costs (excluding hospitalization) per person were estimated at $44.6 (IQR 18.8–92.7). The major contributors of pretreatment costs per person included hospitalization ($67.3), travel expenses ($13.7), chest radiography ($11.2), and medication ($10.8). Factors associated with greater pretreatment costs were residing in a rural area [β = 17.9, (95% CI: 1.07, 34.93)], had ≥ 7 visits to a healthcare provider [β = 19.9, (95% CI: 3.12, 36.74)], and their first TB symptom-related visit was to a private hospital [β = 38.7, (95% CI: 8.20, 69.30)], public hospital [β = 35.7, (95% CI: 8.60, 62.77)] and private primary care [β = 37.9, (95% CI: 19.36, 56.54)], compared to a community health center.

**Conclusions:**

People with DR-TB in Indonesia incurred incremental direct costs during the pretreatment phase. Strengthening TB diagnostic infrastructure and sample referral networks between public and private providers in rural areas is critical to reducing the extra visits and financial burden for DR-TB patients.

## Introduction

Drug-resistant tuberculosis (DR-TB) is a growing global health burden, with an estimated 400,000 new DR-TB cases in 2023 and only 44% of them receiving second-line treatment [1]. Various barriers may prevent patients from obtaining and completing their treatment, including financial burdens. To access proper diagnosis and treatment, DR-TB patients might need to spend 20% or more of their annual household income, which is often reported as catastrophic costs. Although this is not unique to DR-TB patients, based on a study by Akalu et al., DR-TB patients and their families reported much higher catastrophic costs (82%) compared to drug-sensitive TB (DS-TB) patients [2].

Indonesia is among the five countries with the highest estimated DR-TB cases, with only a 56% treatment success rate in 2023 [1,3]. One of the challenges in DR-TB diagnosis and treatment is the limited capacity of TB detection, which leads to delays in treatment, especially among those who reside in rural areas and those with prior visits to private healthcare facilities [4]. As a result, a trend of multiple visits among people with DS-TB and DR-TB emerged in different settings [5,6], as well as in Indonesia [7]. Our previous studies reported that people with TB (PWTB) experienced multiple visits and incurred substantial costs prior to their treatment initiation [8,9]. The National TB Program (NTP) has already provided access to TB diagnosis and treatment; however, out-of-pocket (OOP) expenditures of PWTB remained considerably high [9]. This financial burden is expected to be worse among people with DR-TB, as despite Xpert MTB/RIF’s massive decentralization and rapid expansion in recent years for TB diagnosis in Indonesia [10–12], DR-TB patients still experience diagnostic delays and multiple visits prior to initiating proper treatment [4,10].

Understanding DR-TB patients’ OOP costs to access diagnosis and treatment is necessary to inform public health stakeholders and policymakers in evaluating current financial coverage of TB-related diagnosis and treatment, to achieve better DR-TB diagnosis and treatment initiation. As this information remains unassessed, we therefore aim to calculate pretreatment OOP direct costs among DR-TB patients in West Java, Indonesia, following the national scale-up of Xpert MTB/RIF in 2021. We also aim to identify factors associated with higher OOP costs, to provide a clearer insight into which areas to focus in minimizing financial burden among DR-TB patients.

## Materials and methods

### Study design and setting

This cross-sectional study was conducted in Bandung city, the capital of West Java province, Indonesia. West Java is one of the provinces with the highest TB burden, accounting for an estimated 22% of total national TB cases [13]. In 2023, Bandung city reported an average annual TB notification rate of 434 cases per 100,000 population, and over 300 DR-TB cases were diagnosed and treated [14,15].

Nationwide, diagnosis and treatment for both drug-sensitive TB (DS-TB) and DR-TB follow the National TB Guidelines issued by the Indonesian Ministry of Health. Diagnosis of Rifampicin-resistant TB (RR-TB) is primarily performed using sputum-based rapid molecular testing with Xpert MTB/RIF Ultra. RR-TB diagnosis is carried out by a network of healthcare facilities equipped with Xpert machines, distributed across various healthcare providers in Bandung city. Among 122 total healthcare facilities in Bandung city, the Xpert machines are available in 19 facilities with the following distribution: one provincial health laboratory, one lung clinic, one lung hospital, 11 out of 80 community health centers (CHCs), 3 out of 7 public secondary-level hospitals, one out of 31 private secondary-level hospitals, and one tertiary-level hospital [14,16].

People diagnosed with RR-TB will be referred to a Programmatic Management of Drug-Resistant Tuberculosis (PMDT) treatment center. Before initiating second-line treatment, those patients must undergo a series of baseline medical examinations as required by DR-TB management guidelines [17]. At the time of the study, the main treatment regimens for DR-TB were all oral short treatment regimens (STRs) and long treatment regimens (LTRs) [17]. In 2023, there were two PMDT hospitals and one lung clinic in Bandung city, which served as the main referral sites for DR-TB treatment, with a total of 393 DR-TB patients registered and treated at these sites [15]. All DR-TB diagnosis and treatment were provided by the National TB Program [18].

### Sample size and recruitment

We recruited participants from three recruitment sites comprising one tertiary hospital, one lung hospital, and one lung clinic, at the time of their baseline assessments. We consecutively interviewed participants meeting our eligibility criteria, which were adults with newly diagnosed pulmonary DR-TB and who were in the first six months of their second-line treatment. Participant recruitment was done from 1 February 2023–29 February 2024.

According to the 2022 Indonesian TB Information System (SITB; Sistem Informasi TB) report, a total of 1,800 DR-TB patients were registered in West Java, of which 382 were treated in Bandung. Based on this data, we assumed that 20% of West Java cases were registered in Bandung and used this proportion in our sample size calculation. To achieve 95% power at a 5% significance level, 245 participants would be needed. Accounting for a 5% non-response rate, we aimed to collect 257 subjects, rounding up to 300. According to the SITB report, 60% of Bandung’s RR-TB patients were registered at the tertiary hospital, while 25% and 15% were registered at the lung hospital and lung clinic, respectively. To achieve a representative distribution across all sites, we applied similar percentages to recruit participants, targeting 154 participants from the tertiary hospital, 64 from the lung hospital, and 39 from the lung clinic.

We enrolled all eligible consenting participants until we reached the target sample size. People with a prior history of second-line anti-TB treatment or those who were hospitalized and had communication difficulties due to severe disease symptoms were excluded.

### Data collection

To measure the cost, we collected data using a structured questionnaire based on the Indonesian-adapted version of WHO’s “Tool to Estimate Patient Costs” as previously implemented in studies by Fuady et al. [19] and Lestari et al. [8,9]. Collected data include demographic characteristics, care-seeking behavior, and pretreatment OOP costs. Pretreatment OOP costs included direct medical and non-medical costs. Additional information was collected, including health insurance status, the number and type of facilities visited prior to diagnosis, and coping mechanisms used to manage the existing financial burden (S1 File).

Trained field researchers conducted in-person interviews spanning around 30–45 minutes. The interviewers used specific events as a benchmark. Collected data were recorded on paper-based questionnaires and subsequently transferred to the REDCap database version 14.5.36, with cross-verification by three research assistants. This study was implemented in accordance with relevant health protocols for the use of personal protective equipment (PPE) in standard DR-TB care.

### Definitions

- The pretreatment phase refers to the period of health care visits from the onset of DR-TB-related symptoms to the baseline assessment before treatment initiation [20]. This would cover the whole period where multiple onsets of DR-TB-related symptoms were experienced by patients.
- Direct cost is all OOP expenditures incurred by PWTB and their household/caregivers related to TB seeking diagnosis and treatment, including medical costs and non-medical expenses, minus any reimbursement [20].
- Hospitalization refers to all costs incurred during inpatient care, paid by PWTB and their families/caregivers, including medical costs (e.g., bed-day charges, consultations, laboratory examinations, and supplementary medication) and non-medical costs (e.g., travel and food required during the inpatient period).
- Direct medical costs include OOP payments made after any reimbursement for medical services, such as administrative/consultation fees, diagnostic tests, medications, or other medical procedures. Medical expenses incurred during hospitalization are not included in the direct medical cost calculation.
- Direct non-medical cost is the OOP cost paid for non-medical needs (e.g., transportation, food, accommodation, and other expenses) associated with obtaining TB services, net of any reimbursement. Non-medical expenses related to hospitalization are not included in the direct non-medical cost calculation.
- Coping mechanisms include taking out loans, selling assets, and use of savings to cope with TB [2].

### Data analysis

Descriptive analyses of the data were conducted to summarize the characteristics of DR-TB participants and pretreatment direct costs, including frequencies, percentages, medians, and interquartile ranges (IQRs) stratified by study site. As this study interviewed outpatients, it was expected that only a fraction of participants experienced hospitalization [8,9]. Therefore, we would perform separate calculations with and without hospitalization-related costs included in the total cost calculation. Cost data were recorded and calculated in Indonesian Rupiah (IDR, Rp), and then converted into United States Dollars (USD, $) using the 2023 World Bank midpoint exchange rate of IDR15,236.88 per USD [21].

We used median quantile regression to identify the association between characteristics and higher pretreatment direct costs. The adjusted multivariable model considered age, gender, and insurance status as potential confounders based on prior research [8,9]. Other variables with p-values <0.20 in the univariable analysis were also treated as potential confounders in the adjusted multivariable model [8,9]. Results were presented as coefficients with 95% confidence intervals (CIs), and p-values <0.05 were considered statistically significant. Statistical analyses were performed using STATA version 15.1.

### Ethics statement

This study protocol was approved by the Research Ethics Committee Universitas Padjadjaran Bandung (1345/UN6.KEP/EC/2022) and continuing ethical approval (26/UN6.KEP/EC/2024). All potential participants received written and oral explanations regarding the study prior to giving their consent, and all participants signed a written informed consent before being interviewed.

## Results

### Characteristics of participants

**Fig 1:**
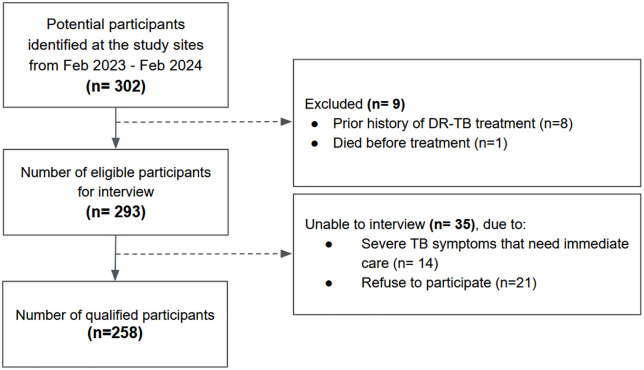
Flowchart of study participants (N = 258). First contact with formal healthcare providers was 63% (n = 163) in public care and 37% (n = 95) in private care. Over half (51.6%; n = 133) had ≥ 7 visits before initiating DR-TB treatment. A total of 129 participants experienced a median delay of 60 days or more from symptom onset to DR-TB diagnosis. Of 129 participants, 76 (59%) initially visited public providers, and 53 (41%) visited private care providers. Additionally, 23.2% (60/258) of participants required hospitalization due to severe disease symptoms prior to second-line treatment.

A total of 302 DR-TB patients were identified from the selected study sites. Of these, 9 (3.0%) did not meet eligibility criteria, and 35 (12%) were unable to attend the interview. Finally, 258 participants were enrolled in this study (Fig 1).

Among the 258 patients enrolled, 132 (51.2%) had a prior history of first-line TB treatment. The median age of participants was 38 years (IQR 27–48), and 57.4% (148/258) were male. The majority of participants resided in rural areas (68.6%; 177/258). Approximately half (49.2%; 127/258) of the participants sought care from informal providers (i.e., drug stores, pharmacies, herbal medicine sellers, independent medical practitioners) prior to visiting formal healthcare providers. More detailed characteristics of the participants are shown in Table 1.

**Table 1:** Sociodemographic, clinical, and health-seeking characteristics of DR-TB participants based on the recruitment sites (N = 258).

Characteristic, n (%)	Tertiary Hospital (n = 150, 58%)	Lung Hospital (n = 65, 25%)	Lung Clinic (n = 43, 17%)
**Gender**			
Male	87 (58)	30 (46.2)	31 (72.1)
Female	63 (42)	35 (53.8)	12 (27.9)
**Age,** median (IQR)	38 (26.25–48)	38 (27–47)	37 (26.5–47)
**Education**			
Below secondary high school	72 (48)	29 (44.6)	19 (44.2)
High school or higher education	78 (52)	36 (55.4)	24 (55.8)
**Residence**			
Urban	35 (23.3)	14 (21.5)	32 (74.4)
Rural	115 (76.7)	51 (78.5)	11 (25.6)
**Treatment Regimen**			
LTR	94 (62.7)	64 (98.5)	37 (86)
STR	26 (17.3)	0 (0)	5 (11.6)
BPaL/M	30 (20)	1 (1.5)	1 (2.3)
**Prior TB history**			
No	73 (48.7)	32 (49.2)	21 (48.8)
Yes	77 (51.3)	33 (50.8)	22 (51.2)
**Comorbid Status**			
No	108 (72)	49 (75.4)	32 (74.4)
Yes	42 (28)	16 (24.6)	11 (25.6)
**Insurance**			
No insurance	60 (40)	16 (24.6)	14 (32.6)
National Health Insurance	90 (60)	49 (75.4)	29 (67.4)
Income (USD)*, median (IQR)	128.6 (0–210)	98.5 (0–223.2)	196.9 (91.9–236.3)
Primary Income Earner: Pretreatment*			
Participant	69 (60)	28 (43.1)	26 (60.5)
Other	46 (40)	37 (56.9)	17 (39.5)
Household Income: Pretreatment*			
Below minimum wage	101 (67.3)	53 (81.5)	31 (72.1)
Above minimum wage	14 (9.3)	12 (18.5)	10 (23.3)
Not available	35 (23.3)	0 (0)	2 (4.7)
**Days from First Symptoms to DR-TB Diagnosis**			
< 60	77 (51.3)	29 (44.6)	23 (53.5)
≥ 60	73 (48.7)	36 (55.4)	20 (46.5)
**Number of visits to healthcare provider before DR-TB treatment initiation**			
< 7	74 (49.3)	32 (49.2)	19 (44.2)
≥ 7	76 (50.7)	33 (50.8)	24 (55.8)
**Type of formal healthcare provider at first visit**			
Community health center	86 (57.3)	32 (49.2)	19 (44.2)
Private primary care	43 (28.7)	16 (24.6)	16 (37.2)
Public hospital	12 (8)	10 (15.4)	4 (9.3)
Private hospital	9 (6)	7 (10.8)	4 (9.3)

*37 participants did not report their income before starting DR-TB treatment. Income was categorized based on the 2023 minimum wage of Bandung City (Rp4,048,500 ~ $265.7).

### Pretreatment direct costs

The calculated median pretreatment direct costs per person (excluding hospitalization costs) were $44.6 (IQR 18.8–92.7). When the calculation was stratified by healthcare facility, participants recruited at the lung hospital reported the highest cost ($83.0, IQR 32.2–120.9) compared to other sites. Of the direct medical costs, chest radiography and medication accounted for the largest share. Transportation was the most frequently reported expense, reported by 99% of all participants.

Only 23.2% (60/258) of participants had experienced hospitalization to a certain extent for TB symptoms prior to their second-line treatment initiation. When hospitalization costs are included, the median pretreatment direct cost across all study sites was $49.0 (IQR 20.6–113.9). With hospitalization included, hospitalization became the highest expense component with a median of $67.3 (IQR 18.5–151.8). Among participants with a hospitalization history, hospitalization-related medical costs contributed 43.2% of their total direct costs, with a median of $62.3 (IQR 31.2–131.3). Details of each cost category for the pretreatment direct cost calculation are shown in Table 2.

**Table 2:** Pretreatment direct costs per person with DR-TB based on the recruitment sites (N = 258).

Cost Category	Tertiary Hospital (n = 150, 58%)		Lung Hospital (n = 65, 25%)		Lung Clinic (n = 43, 17%)	
	n (%)	Median $ (IQR)	n (%)	Median $ (IQR)	n (%)	Median $ (IQR)
Administration	92 (61)	3.9 (1.4–10.0)	43 (66)	6.6 (2.5–16.6)	27 (63)	6.6 (3.0–13.5)
Chest Radiography	46 (31)	10.8 (6.9–14.7)	27 (41)	11.2 (9.8–14.1)	16 (37)	11.5 (7.5–13.4)
Lab Examination^a^	34 (23)	8.2 (5.6–19.2)	16 (25)	11.5 (4.3–13.9)	5 (11)	9.8 (9.8–19.7)
Medication^b^	98 (65)	11.3 (4.6–25.7)	46 (71)	9.2 (4.8–33.6)	31 (72)	9.8 (3.9–29.2)
Travel	148 (99)	12.9 (5.9–26.8)	65 (100)	24.2 (9.8–48.9)	43 (100)	6.2 (4.2–15.1)
Food	138 (92)	6.6 (3.3–17.3)	62 (95)	9.0 (5.7–28.6)	38 (88)	5.8 (2.9–10.9)
Non-medical expenses^c^	16 (11)	0.6 (0.3–1.0)	7 (11)	0.3 (0.3–0.5)	4 (9)	1.0 (0.5–1.5)
**Subtotal cost per person**	**150 (100)**	**38.9 (18.0–83.5)**	**65 (100)**	**83.0 (32.2–120.9)**	**43 (100)**	**33.9 (12.4–77.3)**
Hospitalization	28 (18)	41.4 (18.7–132.9)	23 (35)	77.1 (23.0–212.3)	9 (21)	32.8 (14.8–183.8)
**Total cost per person** (including hospitalization)	**150 (100)**	**40.2 (18.0**–**96.5)**	**65 (100)**	**93.7 (33.9**–**160.5)**	**43 (100)**	**41.2 (13.2**–**91.1)**

IQR = Interquartile range; $ = U.S. Dollar, $1 = 15,236.88 Indonesian rupiah (Source: The World Bank 2023)

^a^Lab examination could include a complete blood test, urine test, and antigen swab test.

^b^Medication included antibiotics or drugs accessed from pharmacies or informal providers.

^c^Other non-medical expenses could include masks and tissue.

### Factors associated with higher costs

Unadjusted analysis showed that location of residence, days from symptom to DR-TB diagnosis (<60 or ≥60), number of visits (<7 or ≥7), and type of formal healthcare facility at the first TB-related visit each had a p-value of <0.2 (Table 3). Hence, these characteristics were considered potential confounders, and the multivariable analyses were adjusted for them, in addition to the literature-based age, gender, and insurance status adjustments.

**Table 3:** Factors associated with median pretreatment direct costs (excluding hospitalization costs) (N = 258).

Characteristics	Median Subtotal Cost (USD)	Unadjusted β (95% CI)	Adjusted* β (95% CI)
**Gender**			
Male	45.2	–	–
Female	44.6	−1.15 (−20.77, 18.47)	−4.65 (−20.60, 11.30)
**Age**, median (IQR)	97.9	0.33 (−0.35, 1.02)	0.03 (−0.54, 0.60)
**Education**			
Below junior high school	44.6	–	–
High school or higher education	45.9	1.31 (−18.20, 20.82)	–
**Residence**			
Urban	28.0	–	–
Rural	48.9	20.94 (0.56, 41.31)	17.99 (1.07, 34.93)**
**Prior TB History**			
No	44.5	–	–
Yes	46.2	1.90 (−17.56, 21.37)	–
**Comorbid Status**			
No	41.7	–	–
Yes	62.3	7.42 (−13.47, 28.30)	–
**Insurance**			
No insurance	46.3	–	–
National Health Insurance	44.5	−2.23 (−21.84, 17.38)	4.58 (−11.89, 21.05)
**Days from First Symptoms to DR-TB Diagnosis**			
< 60	33.9	–	–
≥ 60	56.4	22.51 (5.64, 39.38)	7.71 (−8.13, 23.56)
**Number of visits to healthcare provider before DR-TB treatment initiation**			
< 7	27.3	–	–
≥ 7	62.4	35.05 (17.20, 52.90)	19.93 (3.12, 36.74)**
**Type of formal healthcare provider at first visit**			
Community health center	29.7	–	–
Private primary care	74.0	44.37 (28.53, 60.21)	37.95 (19.36, 56.54)**
Public hospital	65.9	38.86 (15.27, 62.44)	35.68 (8.60, 62.77)**
Private hospital	56.1	28.09 (1.70, 54.50)	38.75 (8.20, 69.30)**

*Adjusted for age, gender, insurance status, and other potential confounders.

**p-value < 0.05.

Factors associated with higher pretreatment direct costs (excluding hospitalization) included residence in rural areas [β = 17.9, (95% CI: 1.07, 34.93)] and having ≥7 visits to healthcare providers before treatment initiation [β = 19.9, (95% CI: 3.12, 36.74)]. Choice of the formal healthcare facility for patients’ first TB symptom-related visit also affected pretreatment direct costs, as first visits to private primary care [β = 37.9, (95% CI: 19.36, 56.54)], public hospital [β = 35.7, (95% CI: 8.60, 62.77)], or private hospital [β = 38.7, (95% CI: 8.20, 69.30)] were associated with higher pretreatment direct costs than visits to a CHC. These associations remained significant after adjusting for age, gender, and other potential confounders, as shown in Table 3.

### Coping mechanisms

Around half (52.5%, 114/258) of the participants borrowed money to cover DR-TB-related costs, with a median amount of $98.5 (IQR 32.8–190.3). Participants primarily obtained these loans from family members (n = 70) or neighbors and relatives (n = 27). The remaining participants obtained loans from cooperatives (n = 8), banks (n = 1), and other sources (n = 8). Forty-four participants sold their personal property (i.e., jewelry, vehicles, and other household items) to raise money, with a median amount of $111 (IQR 67.3–202), to cover their DR-TB-related costs and cope with the impact of their illness.

## Discussion

In this study, we found that DR-TB patients incurred substantial OOP costs during their care-seeking process, with a considerable amount of spending needed for medical-related direct costs. Beyond medical direct costs, travel expenses majorly contributed to the amount of OOP costs spent by DR-TB patients. We also identified that living in rural areas, having more than 7 pretreatment visits, or seeking initial care at public hospitals or private healthcare facilities were significantly associated with higher OOP costs.

This study identified a median pretreatment direct cost of $49.0 among DR-TB patients. This is higher than previous Indonesia-based reports: $37.51 (IQR 20.79–71.25) among pulmonary DS-TB patients in 2017–2019, $27 (IQR 13–62) among MDR-TB patients in 2016, and $33 (IQR 9–64) among DR-TB patients in 2013 [9,22,23]. Cost differences across studies might be influenced by differences in sample recruitment and geographical settings. Additionally, DR-TB patients tend to incur more incremental cost compared to DS-TB patients [6,23–25], which could further explain the difference in our findings with the relatively recent 2017–2019 study on DS-TB patients. Despite differences in the reported amounts, the identified main contributors to pretreatment direct costs across studies were quite consistent with our findings, comprising components related to hospitalization and diagnostic tests [9,22]. Furthermore, a systematic review across various settings showed that medication, chest radiography, and hospitalization costs are among the main drivers of pre-diagnostic costs of TB patients [24], consistent with our findings.

Beyond medical and hospitalization costs, participants in this study largely spent their money travelling to seek TB-related care. This is in line with a previous Indonesian TB-cost study and studies evaluating DR-TB costs among patients in North India and Kazakhstan [22,26], although this is not always the case. A study from Mangalore, India, reported that presumptive DR-TB patients spent more on food compared to travel during their pretreatment phase [27]. Food was also reported as the main non-medical cost driver among Ethiopian and Zimbabwean DR-TB patients [22,28].

In this study, we found that higher pretreatment costs are associated with several factors, including living in rural areas. These patients lived outside Bandung city and could access Bandung city’s facilities by referral or at their own preference, either of which would ultimately lead to additional travel costs. Although rural areas in West Java have been equipped with Xpert CHCs, sample transport is more challenging, and existing rural Xpert CHCs may experience cartridge stockouts or broken modules, a province-wide issue [10]. When Xpert testing is not feasible, presumptive TB patients will need to be examined with sputum microscopy and, potentially, chest radiography [9,29], which would increase the number of visits and travel costs. If the additional tests are done in private healthcare facilities, they may incur higher costs, as some private facilities are not included in the NHI network or the services exceed NHI’s reimbursement limit.

Our analysis also showed that patients chose for their first TB-related visit at private primary care facilities had the highest pretreatment direct costs compared with the rest of the participants. This is partially consistent with a previous study from Indonesia, which found that patients who first sought care in the private sector incurred higher pretreatment costs than those who initiated care at public hospitals or community health centers [8]. This may be attributed to the perception that seeking care at private care is considered more convenient and accessible, despite higher medical costs [30,31]. However, our study found that patients who opted for public hospitals incurred more costs than private hospitals. This difference was largely driven by non-medical spending, with travel and food costs particularly high among participants who visited public hospitals as their first care providers. This finding is similar to an Indian study that found visiting public health facilities incurred higher non-medical costs, despite lower medical expenses [26]. In our setting, the availability of DR-TB diagnostic and treatment services is concentrated in district centers, which are often accessible through multiple referrals requiring far and expensive travel, especially for those residing in rural areas, which are represented by the majority of our participants.

This study has several limitations. First, all cost data were self-reported by patients through in-person interviews after they had already arrived at PMDT centers for baseline assessment; hence, they are subject to recall bias. This bias could be more influential in hospitalization-related costs, where costs incurred by caregivers were also taken into account. However, we tried to minimize recall bias by recruiting participants who had started their treatment no more than 6 months before the interview. Second, of 21 patients that were unable to attend the interview, 12/21 were male, and 18/21 resided in rural areas. As the majority of them fell within these specific demographic characteristics, selection bias may have affected the study. However, we have recruited a sufficient number of participants, and we found that males as well as rural residents dominated the study participants, suggesting that the bias from the non-participation of the 21 patients could be negligible. Third, we did not present catastrophic costs in this study, as many patients are still on ongoing treatments. Future studies would need to analyze catastrophic costs by doing follow-up towards the DR-TB patients until they have completed treatment and collect data on their direct and indirect costs during treatment. However, this study is among the few in Indonesia to specifically focus on direct OOP costs by DR-TB patients, offering valuable insights into the financial burden during the DR-TB pretreatment phase following the national expansion of Xpert MTB/RIF in Indonesia in 2021.

## Conclusions

This study highlights that despite the NTP covering DR-TB diagnosis and treatment medications, people with DR-TB still suffer significant OOP costs during the pretreatment phase after Indonesia’s national expansion of Xpert MTB/RIF, emphasizing the presence of financial burden among DR-TB patients in a low-resource setting. Based on our findings, stronger DR-TB diagnostic infrastructure and more efficient clinical sample referral mechanisms are needed among public and private providers that cover less developed areas. These measures should expand access to rapid molecular testing and reduce unnecessary visits prior to diagnosis and treatment. Addressing the issues highlighted in this study can alleviate some of the burden endured by DR-TB patients, which could eventually lead to improvement to DR-TB diagnosis and treatment initiation.

## Supporting information

S1 File Pretreatment costs questionnaire. (PDF)
