# Supplementary material for: Pretreatment out-of-pocket costs for people with drug-resistant tuberculosis in Bandung, Indonesia
Source: PLoS One. 2026 Jul 1;21(7):e0352658. doi: 10.1371/journal.pone.0352658 (PMC13322529; doi:10.1371/journal.pone.0352658)
Supplement: S1 File — (PDF) [file pone.0352658.s001.pdf]

DR-TB and DM Survey of patient pathway and patient costs for DR-TB treatment	Research site ID: I__I – I__I__I__I	Participant ID: I__I__I__I__I__I__I__I	Interview date (Day/month/year) _____/_____/______	Interviewer's initials

Part I. Patient Information (if possible, data should be obtained from SITB prior to interview & confirmed during interview)

General information	
**1. Full name**	
**2. Medical record number/SITB** (filled in by the enumerator)	
**3. Gender**	☐ Female ☐ Male
**4. Age of the patient**	________year
**5. Address (based on KTP)**	
**b) Classification** (filled in by the enumerator)	☐ Urban<br>☐ Rural
**6. Place of residence for the last 6 months**	
**b) Classification** (filled in by the enumerator)	☐ Urban<br>☐ Rural
**7. Highest education level**	☐ No schooling<br>☐ Elementary school/equivalent<br>☐ Junior high school/equivalent<br>☐ High school/equivalent<br>☐ Higher/vocational education<br>☐ Others (specify): ________________
**8. Do you have BPJS?**	☐ Yes<br>☐ No
**9. Do you seek treatment using BPJS?**	☐ Yes<br>☐ No
**10. What is your current job?**	1. Formal sector jobs<br>2. Informal sector jobs<br>3. No job yet/looking for work<br>4. Retired<br>5. Housewife<br>6. Student / student<br>7. Other (specify) ___________________
**11. How much do you earn per month?**	Rp_________

Disease History	
**12. Date of TB diagnosis**<br>*(in day/month/year)*	____/____/_____
**13. Current TB treatment start date**<br>*(in day/month/year)*	_____/____/______
**14. TB treatment regimen given**	☐ Short *-term regiment* (STR)<br>☐ *Long-term* regiment (LTR)

**1 5. Were you informed about the duration of the treatment you received?**	**Intensive phase: __________**months<br>**Continuation phase: __________**months
**16. Current phase of TB management**	□ Intensive phase, completed _______days<br>□ Continuation phase, completed _______days
**17. Have you ever received TB treatment before?**	□ Yes<br>□ No *(Go to Q18)*
**b) If yes, did you complete your previous TB treatment?**	□ Yes<br>□ No
**c) If not completing treatment, why?**	□ Medication side effects<br>□ Lack of money for medical treatment<br>□ Distance to health facility<br>□ Others (specify): ________
**18. History of diabetes mellitus (DM)**	□ Yes □ No *(Go to P22)*
**19. Time of DM diagnosis**<br>*(in day/month/year)*	_____/____/______
**20. DM treatment start time**<br>*(in day/month/year)*	_____/____/______
**b) Drugs used for the current treatment of DM**<br>*(check all that apply)*	□ Oral hypoglycemic drugs (OHO) □ Insulin<br>□ Others (specify) __________________
**c) Are DM drugs changed after DR-TB diagnosis?**	□ Yes □ No<br>□ Newly diagnosed DM patient (*Go to P21)*
**d) If yes, what DM medication did you use before being diagnosed with DR-TB?**	□ Oral hypoglycemic drugs (OHO) □ Insulin<br>□ Others (specify) __________________
**21. Location of current management of DM** *(circle one)*	1. General practitioner in private practice<br>2. Private practice specialist doctor<br>3. Community Health Centers, ________________<br>4. Private hospital<br>5. Lung Clinic<br>6. Lung Hospital<br>7. Regional public hospital, write the name ____________<br>8. Tertiary hospital<br>9. Other (specify) ______________
**b) Sectoral affiliation of DM management facilities**<br>(filled in by the enumerator)	□ Government □ Private □ Religious organizations<br>□ NGOs/CSOs □ Other (specify) ________________
**c) Blood sugar levels at *baseline***<br>*(in numbers, from SITB)*	1. HbA1c ____<br>2. GDS ______
**22. Do you have any other illnesses?**<br>*(more than one answer can be chosen, to be confirmed with data from SITB)*	1. HIV □ Yes □ No<br>2. Hypertension □ Yes □ No<br>3. Dyslipidemia □ Yes □ No<br>4. Heart disease □ Yes □ No<br>5. Others, specify ______

Part II. TB treatment services to DR-TB diagnosis

**Treatment seeking behavior**	
**23. What symptoms did you experience that made you decide to go for treatment for your current illness?**	□ Cough<br>□ Night sweats

In patients diagnosed with DR-TB after failing DS-TB treatment, the symptoms in question are the symptoms the patient feels when the patient's treatment is declared a failure.	☐ Bloody cough<br>☐ Weight loss<br>☐ Other (specify) ____________
**2 4. When did you first experience the symptoms of TB that prompted you to seek treatment?**<br>(example: cough, cold sweat, chest pain, fatigue, fever, weight loss) *(in days/month/year)*<br><br>*In patients diagnosed with DR-TB after failing DS-TB treatment, the symptoms in question are the symptoms the patient feels when the patient's treatment is declared a failure.*	_____/____/_____
**25. When did you first visit your health care provider regarding your DR-TB symptoms?**<br>*(in day/month/year)*<br><br>*In patients diagnosed with DR-TB after failing DS-TB treatment, the symptoms in question are the symptoms the patient feels when the patient's treatment is declared a failure.*	_____/____/_____
**26. What was the pattern/timeline of your visit to the health care provider from the first time you came to being diagnosed with DR-TB?**<br>List all health care providers you see. Check one option per column and fill in the detailed information about each service provider. **Please circle the health care provider where you were diagnosed with DR-TB**.	

No	Type of service provider (circle the appropriate one)	Hospital sector affiliation (circle the appropriate ONE)	Examinations examined at this service provider (check ALL that are appropriate, ask if the patient has a photo of the results of the examination)	Medicines given by this service provider (tick ALL that apply)	Reason for visit to this service provider (tick ALL that apply)	Follow-up plan by healthcare provider (check ALL that apply)
1	1. Pharmacy<br>2. Drug store<br>3. Traditional/alternative/herbal medicine<br>4. General practitioner in private practice<br>5. Specialist in private practice<br>6. Public health center, write the name ___________<br>7. Clinic, write the name ___________<br>8. Private hospital, write the name _______________<br>9. BBKPM Bandung<br>10. Rotinsulu Pulmonary Hospital<br>1 1. Regional general hospital, write the name ___________<br>1 2. RSUP Dr. Hasan Sadikin<br>1 3. Others (please specify)	1. Government<br>2. Private 3. Religious organization 4. NGOs/Ormas5. Other______	□ None<br>□ Sputum examination □ X-ray/other imaging □ Blood test<br>□ Others (specify) __________________	□ None<br>□ Pain relief<br>□ Cough reliever<br>□ Antibiotics, specify _______<br>□ Corticosteroids<br>□ Herbal medicine<br>□ DM medicine<br>□ Can't remember the medicine<br>□ Other (specify) _______________	□ Referral by previous service provider<br>□ Free/affordable<br>□ Seek treatment for DM at this service provider<br>□ Have had treatment with this service provider (for reasons other than DM)<br>□ Distance/easy access<br>□ Recommended by relatives, friends, or colleagues<br>□ Medication available at these providers<br>□ Diagnostic tests are available at these providers<br>□ Diagnostic tests & medications are available at the same providers<br>□ Mild symptoms<br>□ Feels that the symptoms you are experiencing are related to witchcraft or the occult<br>□ Worried about the severity of your symptoms<br>□ Believes service provider expertise<br>□ Others (specify) ________________	□<br>Refer for diagnosis<br>□ Refer for management<br>□ Repeat visit for treatment<br>□ No follow up planned/I refer myself for next visit

No	Type of service provider (circle the appropriate one)	Hospital sector affiliation (circle the appropriate ONE)	Examinations examined at this service provider (check ALL that are appropriate, ask if the patient has a photo of the results of the examination)	Medicines given by this service provider (tick ALL that apply)	Reason for visit to this service provider (tick ALL that apply)	Follow-up plan by healthcare provider (check ALL that apply)
2	1. Pharmacy<br>2. Drug store3. Traditional/alternative/herbal medicine<br>4. General practitioner in private practice<br>5. Specialist in private practice<br>6. Public health center, write the name ___________<br>7. Clinic, write the name<br>___________<br>8. Private hospital, write the name _______________<br>9. BBKPM Bandung<br>10. Rotinsulu Pulmonary Hospital<br>1 1. Regional general hospital, write the name ___________<br>1 2. RSUP Dr. Hasan Sadikin<br>1 3. Others (please specify)	1. Government<br>2. Private<br>3. Religious organization<br>4. NGOs/Ormas<br>5. Other____	□ None<br>□ Sputum examination □ X-ray/other imaging □ Blood test □ Other (specify)<br>______<br>□ Microscopic examination of sputum smear<br>□ Culture<br>□ Drug sensitivity test □ GeneXpert<br>□ Others (specify)<br>__________________	□ None<br>□ Pain relief<br>□ Cough reliever<br>□ Antibiotics, specify<br>_______<br>□ Corticosteroids<br>□ Herbal medicine<br>□ DM medicine<br>□ Can't remember the medicine<br>□ Other (specify)<br>_______________	□ Referral by previous service provider<br>□ Free/affordable<br>□ Seek treatment for DM at this service provider<br>□ Have had treatment with this service provider (for reasons other than DM)<br>□ Distance/easy access<br>□ Recommended by relatives, friends, or colleagues<br>□ Medication available at these providers<br>□ Diagnostic tests are available at these providers<br>□ Diagnostic tests & medications are available at the same providers<br>□ Mild symptoms<br>□ Feels that the symptoms you are experiencing are related to witchcraft or the occult<br>□ Worried about the severity of your symptoms<br>□ Believes service provider expertise<br>□ Others (specify) ________________	□<br>Refer for diagnosis<br>□ Refer for management<br>□ Repeat visit for treatment<br>□ No follow up planned/I refer myself for next visit
3	1. Pharmacy<br>2. Drug store3. Traditional/alternative/herbal medicine<br>4. General practitioner in private practice<br>5. Specialist in private practice<br>6. Public health center, write the name ___________<br>7. Clinic, write the name<br>___________<br>8. Private hospital, write the name _______________<br>9. BBKPM Bandung<br>10. Rotinsulu Pulmonary	1. Government<br>2. Private<br>3. Religious organization<br>4. NGOs/Ormas<br>5. Other____	□ None<br>□ Sputum examination □ X-ray/other imaging □ Blood test □ Other (specify)<br>______<br>□ Microscopic examination of sputum smear<br>□ Culture<br>□ Drug sensitivity test □ GeneXpert<br>□ Others (specify)<br>__________________	□ None<br>□ Pain relief<br>□ Cough reliever<br>□ Antibiotics, specify<br>_______<br>□ Corticosteroids<br>□ Herbal medicine<br>□ DM medicine<br>□ Can't remember the medicine<br>□ Other (specify)<br>_______________	□ Referral by previous service provider<br>□ Free/affordable<br>□ Seek treatment for DM at this service provider<br>□ Have had treatment with this service provider (for reasons other than DM)<br>□ Distance/easy access<br>□ Recommended by relatives, friends, or colleagues<br>□ Medication available at these providers<br>□ Diagnostic tests are available at these providers<br>□ Diagnostic tests & medications are available at the same providers<br>□ Mild symptoms	□<br>Refer for diagnosis<br>□ Refer for management<br>□ Repeat visit for treatment<br>□ No follow up planned/I refer myself for next visit

No	Type of service provider (circle the appropriate one)	Hospital sector affiliation (circle the appropriate ONE)	Examinations examined at this service provider (check ALL that are appropriate, ask if the patient has a photo of the results of the examination)	Medicines given by this service provider (tick ALL that apply)	Reason for visit to this service provider (tick ALL that apply)	Follow-up plan by healthcare provider (check ALL that apply)
	Hospital<br>1 1. Regional general hospital, write the name ___________<br>1 2. RSUP Dr. Hasan Sadikin<br>1 3. Others (please specify)				▯ Feels that the symptoms you are experiencing are related to witchcraft or the occult<br>▯ Worried about the severity of your symptoms<br>▯ Believes service provider expertise<br>▯ Others (specify) ________________	
4	1. Pharmacy<br>2. Drug store3. Traditional/alternative/herbal medicine<br>4. General practitioner in private practice<br>5. Specialist in private practice<br>6. Public health center, write the name ___________<br>7. Clinic, write the name ___________<br>8. Private hospital, write the name _______________<br>9. BBKPM Bandung<br>10. Rotinsulu Pulmonary Hospital<br>1 1. Regional general hospital, write the name ___________<br>1 2. RSUP Dr. Hasan Sadikin<br>1 3. Others (please specify)	1. Government<br>2. Private<br>3. Religious organization<br>4. NGOs/Ormas<br>5. Other____	▯ None<br>▯ Sputum examination □ X-ray/other imaging □ Blood test □ Other (specify) ______<br>▯ Microscopic examination of sputum smear<br>▯ Culture<br>▯ Drug sensitivity test □ GeneXpert<br>▯ Others (specify) __________________	▯ None<br>▯ Pain relief<br>▯ Cough reliever<br>▯ Antibiotics, specify _______<br>▯ Corticosteroids<br>▯ Herbal medicine<br>▯ DM medicine<br>▯ Can't remember the medicine<br>▯ Other (specify) _______________	▯ Referral by previous service provider<br>▯ Free/affordable<br>▯ Seek treatment for DM at this service provider<br>▯ Have had treatment with this service provider (for reasons other than DM)<br>▯ Distance/easy access<br>▯ Recommended by relatives, friends, or colleagues<br>▯ Medication available at these providers<br>▯ Diagnostic tests are available at these providers<br>▯ Diagnostic tests & medications are available at the same providers<br>▯ Mild symptoms<br>▯ Feels that the symptoms you are experiencing are related to witchcraft or the occult<br>▯ Worried about the severity of your symptoms<br>▯ Believes service provider expertise<br>▯ Others (specify) ________________	▯<br>Refer for diagnosis<br>▯ Refer for management<br>▯ Repeat visit for treatment<br>▯ No follow up planned/I refer myself for next visit

No	Type of service provider (circle the appropriate one)	Hospital sector affiliation (circle the appropriate ONE)	Examinations examined at this service provider (check ALL that are appropriate, ask if the patient has a photo of the results of the examination)	Medicines given by this service provider (tick ALL that apply)	Reason for visit to this service provider (tick ALL that apply)	Follow-up plan by healthcare provider (check ALL that apply)
5	1. Pharmacy<br>2. Drug store3. Traditional/alternative/herbal medicine<br>4. General practitioner in private practice<br>5. Specialist in private practice<br>6. Public health center, write the name ___________<br>7. Clinic, write the name ___________<br>8. Private hospital, write the name _______________<br>9. BBKPM Bandung<br>10. Rotinsulu Pulmonary Hospital<br>1 1. Regional general hospital, write the name ___________<br>1 2. RSUP Dr. Hasan Sadikin<br>1 3. Others (please specify)	1. Government<br>2. Private<br>3. Religious organization<br>4. NGOs/Ormas<br>5. Other____	□ None<br>□ Sputum examination □ X-ray/other imaging □ Blood test □ Other (specify) ______<br>□ Microscopic examination of sputum smear<br>□ Culture<br>□ Drug sensitivity test □ GeneXpert<br>□ Others (specify) __________________	□ None<br>□ Pain relief<br>□ Cough reliever<br>□ Antibiotics, specify _______<br>□ Corticosteroids<br>□ Herbal medicine<br>□ DM medicine<br>□ Can't remember the medicine<br>□ Other (specify) _______________	□ Referral by previous service provider<br>□ Free/affordable<br>□ Seek treatment for DM at this service provider<br>□ Have had treatment with this service provider (for reasons other than DM)<br>□ Distance/easy access<br>□ Recommended by relatives, friends, or colleagues<br>□ Medication available at these providers<br>□ Diagnostic tests are available at these providers<br>□ Diagnostic tests & medications are available at the same providers<br>□ Mild symptoms<br>□ Feels that the symptoms you are experiencing are related to witchcraft or the occult<br>□ Worried about the severity of your symptoms<br>□ Believes service provider expertise<br>□ Others (specify) ________________	□<br>Refer for diagnosis<br>□ Refer for management<br>□ Repeat visit for treatment<br>□ No follow up planned/I refer myself for next visit
6	1. Pharmacy<br>2. Drug store3. Traditional/alternative/herbal medicine<br>4. General practitioner in private practice<br>5. Specialist in private practice<br>6. Public health center, write the name ___________<br>7. Clinic, write the name ___________<br>8. Private hospital, write the name _______________<br>9. BBKPM Bandung<br>10. Rotinsulu Pulmonary	1. Government<br>2. Private<br>3. Religious organization<br>4. NGOs/Ormas<br>5. Other____	□ None<br>□ Sputum examination □ X-ray/other imaging □ Blood test □ Other (specify) ______<br>□ Microscopic examination of sputum smear<br>□ Culture<br>□ Drug sensitivity test □ GeneXpert<br>□ Others (specify) __________________	□ None<br>□ Pain relief<br>□ Cough reliever<br>□ Antibiotics, specify _______<br>□ Corticosteroids<br>□ Herbal medicine<br>□ DM medicine<br>□ Can't remember the medicine<br>□ Other (specify) _______________	□ Referral by previous service provider<br>□ Free/affordable<br>□ Seek treatment for DM at this service provider<br>□ Have had treatment with this service provider (for reasons other than DM)<br>□ Distance/easy access<br>□ Recommended by relatives, friends, or colleagues<br>□ Medication available at these providers<br>□ Diagnostic tests are available at these providers<br>□ Diagnostic tests & medications are available at the same providers<br>□ Mild symptoms	□<br>Refer for diagnosis<br>□ Refer for management<br>□ Repeat visit for treatment<br>□ No follow up planned/I refer myself for next visit

No	Type of service provider (circle the appropriate one)	Hospital sector affiliation (circle the appropriate ONE)	Examinations examined at this service provider (check ALL that are appropriate, ask if the patient has a photo of the results of the examination)	Medicines given by this service provider (tick ALL that apply)	Reason for visit to this service provider (tick ALL that apply)	Follow-up plan by healthcare provider (check ALL that apply)
	Hospital<br>1 1. Regional general hospital, write the name __________<br>1 2. RSUP Dr. Hasan Sadikin<br>1 3. Others (please specify)				Feels that the symptoms you are experiencing are related to witchcraft or the occult<br>Worried about the severity of your symptoms<br>Believes service provider expertise<br>Others (specify)_______	
7	1. Pharmacy<br>2. Drug store3. Traditional/alternative/herbal medicine<br>4. General practitioner in private practice<br>5. Specialist in private practice<br>6. Public health center, write the name __________<br>7. Clinic, write the name __________<br>8. Private hospital, write the name ______________<br>9. BBKPM Bandung<br>10. Rotinsulu Pulmonary Hospital<br>1 1. Regional general hospital, write the name __________<br>1 2. RSUP Dr. Hasan Sadikin<br>1 3. Others (please specify)	1. Government<br>2. Private<br>3. Religious organization<br>4. NGOs/Ormas<br>5. Other____	None<br>Sputum examination □ X-ray/other imaging □ Blood test □ Other (specify) ______<br>Microscopic examination of sputum smear<br>Culture<br>Drug sensitivity test □ GeneXpert<br>Others (specify) _________________	None<br>Pain relief<br>Cough reliever<br>Antibiotics, specify _______<br>Corticosteroids<br>Herbal medicine<br>DM medicine<br>Can't remember the medicine<br>Other (specify) ______________	Referral by previous service provider<br>Free/affordable<br>Seek treatment for DM at this service provider<br>Have had treatment with this service provider (for reasons other than DM)<br>Distance/easy access<br>Recommended by relatives, friends, or colleagues<br>Medication available at these providers<br>Diagnostic tests are available at these providers<br>Diagnostic tests & medications are available at the same providers<br>Mild symptoms<br>Feels that the symptoms you are experiencing are related to witchcraft or the occult<br>Worried about the severity of your symptoms<br>Believes service provider expertise<br>Others (specify) _______________	Refer for diagnosis<br>Refer for management<br>Repeat visit for treatment<br>No follow up planned/I refer myself for next visit

No	Type of service provider (circle the appropriate one)	Hospital sector affiliation (circle the appropriate ONE)	Examinations examined at this service provider (check ALL that are appropriate, ask if the patient has a photo of the results of the examination)	Medicines given by this service provider (tick ALL that apply)	Reason for visit to this service provider (tick ALL that apply)	Follow-up plan by healthcare provider (check ALL that apply)
8	1. Pharmacy<br>2. Drug store3. Traditional/alternative/herbal medicine<br>4. General practitioner in private practice<br>5. Specialist in private practice<br>6. Public health center, write the name ___________<br>7. Clinic, write the name ___________<br>8. Private hospital, write the name _______________<br>9. BBKPM Bandung<br>10. Rotinsulu Pulmonary Hospital<br>1 1. Regional general hospital, write the name ___________<br>1 2. RSUP Dr. Hasan Sadikin<br>1 3. Others (please specify)	1. Government<br>2. Private<br>3. Religious organization<br>4. NGOs/Ormas<br>5. Other____	□ None<br>□ Sputum examination □ X-ray/other imaging □ Blood test □ Other (specify) ______<br>□ Microscopic examination of sputum smear<br>□ Culture<br>□ Drug sensitivity test □ GeneXpert<br>□ Others (specify) ___________________	□ None<br>□ Pain relief<br>□ Cough reliever<br>□ Antibiotics, specify _______<br>□ Corticosteroids<br>□ Herbal medicine<br>□ DM medicine<br>□ Can't remember the medicine<br>□ Other (specify) _______________	□ Referral by previous service provider<br>□ Free/affordable<br>□ Seek treatment for DM at this service provider<br>□ Have had treatment with this service provider (for reasons other than DM)<br>□ Distance/easy access<br>□ Recommended by relatives, friends, or colleagues<br>□ Medication available at these providers<br>□ Diagnostic tests are available at these providers<br>□ Diagnostic tests & medications are available at the same providers<br>□ Mild symptoms<br>□ Feels that the symptoms you are experiencing are related to witchcraft or the occult<br>□ Worried about the severity of your symptoms<br>□ Believes service provider expertise<br>□ Others (specify)	□ Refer for diagnosis<br>□ Refer for management<br>□ Repeat visit for treatment<br>□ No follow up planned/I refer myself for next visit

No	Type of service provider (circle the appropriate one)	Hospital sector affiliation (circle the appropriate ONE)	Examinations examined at this service provider (check ALL that are appropriate, ask if the patient has a photo of the results of the examination)	Medicines given by this service provider (tick ALL that apply)	Reason for visit to this service provider (tick ALL that apply)	Follow-up plan by healthcare provider (check ALL that apply)
9	1. Pharmacy<br>2. Drug store3. Traditional/alternative/herbal medicine<br>4. General practitioner in private practice<br>5. Specialist in private practice<br>6. Public health center, write the name __________<br>7. Clinic, write the name __________<br>8. Private hospital, write the name ______________<br>9. BBKPM Bandung<br>10. Rotinsulu Pulmonary Hospital<br>1 1. Regional general hospital, write the name __________<br>1 2. RSUP Dr. Hasan Sadikin<br>1 3. Others (please specify)	1. Government<br>2. Private<br>3. Religious organization<br>4. NGOs/Ormas<br>5. Other____	□ None<br>□ Sputum examination □ X-ray/other imaging □ Blood test □ Other (specify) ______<br>□ Microscopic examination of sputum smear<br>□ Culture<br>□ Drug sensitivity test □ GeneXpert<br>□ Others (specify) _________________	□ None<br>□ Pain relief<br>□ Cough reliever<br>□ Antibiotics, specify _______<br>□ Corticosteroids<br>□ Herbal medicine<br>□ DM medicine<br>□ Can't remember the medicine<br>□ Other (specify) ______________	□ Referral by previous service provider<br>□ Free/affordable<br>□ Seek treatment for DM at this service provider<br>□ Have had treatment with this service provider (for reasons other than DM)<br>□ Distance/easy access<br>□ Recommended by relatives, friends, or colleagues<br>□ Medication available at these providers<br>□ Diagnostic tests are available at these providers<br>□ Diagnostic tests & medications are available at the same providers<br>□ Mild symptoms<br>□ Feels that the symptoms you are experiencing are related to witchcraft or the occult<br>□ Worried about the severity of your symptoms<br>□ Believes service provider expertise<br>□ Others (specify) _______________	□<br>Refer for diagnosis<br>□ Refer for management<br>□ Repeat visit for treatment<br>□ No follow up planned/I refer myself for next visit

No	Type of service provider (circle the appropriate one)	Hospital sector affiliation (circle the appropriate ONE)	Examinations examined at this service provider (check ALL that are appropriate, ask if the patient has a photo of the results of the examination)	Medicines given by this service provider (tick ALL that apply)	Reason for visit to this service provider (tick ALL that apply)	Follow-up plan by healthcare provider (check ALL that apply)
10	1. Pharmacy<br>2. Drug store3. Traditional/alternative/herbal medicine<br>4. General practitioner in private practice<br>5. Specialist in private practice<br>6. Public health center, write the name ___________<br>7. Clinic, write the name ___________<br>8. Private hospital, write the name _______________<br>9. BBKPM Bandung<br>10. Rotinsulu Pulmonary Hospital<br>1 1. Regional general hospital, write the name ___________<br>1 2. RSUP Dr. Hasan Sadikin<br>1 3. Others (please specify)	1. Government<br>2. Private<br>3. Religious organization<br>4. NGOs/Ormas<br>5. Other____	None<br>Sputum examination □ X-ray/other imaging □ Blood test □ Other (specify) ______<br>Microscopic examination of sputum smear<br>Culture<br>Drug sensitivity test □ GeneXpert<br>Others (specify) __________________	None<br>Pain relief<br>Cough reliever<br>Antibiotics, specify _______<br>Corticosteroids<br>Herbal medicine<br>DM medicine<br>Can't remember the medicine<br>Other (specify) _______________	Referral by previous service provider<br>Free/affordable<br>Seek treatment for DM at this service provider<br>Have had treatment with this service provider (for reasons other than DM)<br>Distance/easy access<br>Recommended by relatives, friends, or colleagues<br>Medication available at these providers<br>Diagnostic tests are available at these providers<br>Diagnostic tests & medications are available at the same providers<br>Mild symptoms<br>Feels that the symptoms you are experiencing are related to witchcraft or the occult<br>Worried about the severity of your symptoms<br>Believes service provider expertise<br>Others (specify) ________________	Refer for diagnosis<br>Refer for management<br>Repeat visit for treatment<br>No follow up planned/I refer myself for next visit
11	1. Pharmacy<br>2. Drug store3. Traditional/alternative/herbal medicine<br>4. General practitioner in private practice<br>5. Specialist in private practice<br>6. Public health center, write the name ___________<br>7. Clinic, write the name ___________<br>8. Private hospital, write the name _______________<br>9. BBKPM Bandung	1. Government<br>2. Private<br>3. Religious organization<br>4. NGOs/Ormas<br>5. Other____	None<br>Sputum examination □ X-ray/other imaging □ Blood test □ Other (specify) ______<br>Microscopic examination of sputum smear<br>Culture<br>Drug sensitivity test □ GeneXpert<br>Others (specify) __________________	None<br>Pain relief<br>Cough reliever<br>Antibiotics, specify _______<br>Corticosteroids<br>Herbal medicine<br>DM medicine<br>Can't remember the medicine<br>Other (specify) _______________	Referral by previous service provider<br>Free/affordable<br>Seek treatment for DM at this service provider<br>Have had treatment with this service provider (for reasons other than DM)<br>Distance/easy access<br>Recommended by relatives, friends, or colleagues<br>Medication available at these providers<br>Diagnostic tests are available at these providers<br>Diagnostic tests & medications are available at the same providers	Refer for diagnosis<br>Refer for management<br>Repeat visit for treatment<br>No follow up planned/I refer myself for next visit

No	Type of service provider (circle the appropriate one)	Hospital sector affiliation (circle the appropriate ONE)	Examinations examined at this service provider (check ALL that are appropriate, ask if the patient has a photo of the results of the examination)	Medicines given by this service provider (tick ALL that apply)	Reason for visit to this service provider (tick ALL that apply)	Follow-up plan by healthcare provider (check ALL that apply)
	10 . Rotinsulu Pulmonary Hospital<br>1 1. Regional general hospital, write the name __________<br>1 2. Dr. Hasan Sadikin<br>1 3. Others (please specify)				□ Mild symptoms<br>□ Feels that the symptoms you are experiencing are related to witchcraft or the occult<br>□ Worried about the severity of your symptoms<br>□ Believes service provider expertise<br>□ Others (specify)	
12	1. Pharmacy<br>2. Drug store3. Traditional/alternative/herbal medicine<br>4. General practitioner in private practice<br>5. Specialist in private practice<br>6. Public health center, write the name ___________<br>7. Clinic, write the name ___________<br>8. Private hospital, write the name _______________<br>9. BBKPM Bandung<br>10. Rotinsulu Pulmonary Hospital<br>1 1. Regional general hospital, write the name __________<br>1 2. RSUP Dr. Hasan Sadikin<br>1 3. Others (please specify)	1. Government<br>2. Private<br>3. Religious organization<br>4. NGOs/Ormas<br>5. Other____	□ None<br>□ Sputum examination □ X-ray/other imaging □ Blood test □ Other (specify) ______<br>□ Microscopic examination of sputum smear<br>□ Culture<br>□ Drug sensitivity test □ GeneXpert<br>□ Others (specify) _________________	□ None<br>□ Pain relief<br>□ Cough reliever<br>□ Antibiotics, specify _______<br>□ Corticosteroids<br>□ Herbal medicine<br>□ DM medicine<br>□ Can't remember the medicine<br>□ Other (specify) ______________	□ Referral by previous service provider<br>□ Free/affordable<br>□ Seek treatment for DM at this service provider<br>□ Have had treatment with this service provider (for reasons other than DM)<br>□ Distance/easy access<br>□ Recommended by relatives, friends, or colleagues<br>□ Medication available at these providers<br>□ Diagnostic tests are available at these providers<br>□ Diagnostic tests & medications are available at the same providers<br>□ Mild symptoms<br>□ Feels that the symptoms you are experiencing are related to witchcraft or the occult<br>□ Worried about the severity of your symptoms<br>□ Believes service provider expertise<br>□ Others (specify) ________________	□ Refer for diagnosis<br>□ Refer for management<br>□ Repeat visit for treatment<br>□ No follow up planned/I refer myself for next visit

No	Type of service provider (circle the appropriate one)	Hospital sector affiliation (circle the appropriate ONE)	Examinations examined at this service provider (check ALL that are appropriate, ask if the patient has a photo of the results of the examination)	Medicines given by this service provider (tick ALL that apply)	Reason for visit to this service provider (tick ALL that apply)	Follow-up plan by healthcare provider (check ALL that apply)
13	1. Pharmacy<br>2. Drug store3. Traditional/alternative/herbal medicine<br>4. General practitioner in private practice<br>5. Specialist in private practice<br>6. Public health center, write the name ___________<br>7. Clinic, write the name ___________<br>8. Private hospital, write the name _______________<br>9. BBKPM Bandung<br>10. Rotinsulu Pulmonary Hospital<br>1 1. Regional general hospital, write the name ___________<br>1 2. RSUP Dr. Hasan Sadikin<br>1 3. Others (please specify)	1. Government<br>2. Private<br>3. Religious organization<br>4. NGOs/Ormas<br>5. Other____	□ None<br>□ Sputum examination □ X-ray/other imaging □ Blood test □ Other (specify) ______<br>□ Microscopic examination of sputum smear<br>□ Culture<br>□ Drug sensitivity test □ GeneXpert<br>□ Others (specify) __________________	□ None<br>□ Pain relief<br>□ Cough reliever<br>□ Antibiotics, specify _______<br>□ Corticosteroids<br>□ Herbal medicine<br>□ DM medicine<br>□ Can't remember the medicine<br>□ Other (specify) _______________	□ Referral by previous service provider<br>□ Free/affordable<br>□ Seek treatment for DM at this service provider<br>□ Have had treatment with this service provider (for reasons other than DM)<br>□ Distance/easy access<br>□ Recommended by relatives, friends, or colleagues<br>□ Medication available at these providers<br>□ Diagnostic tests are available at these providers<br>□ Diagnostic tests & medications are available at the same providers<br>□ Mild symptoms<br>□ Feels that the symptoms you are experiencing are related to witchcraft or the occult<br>□ Worried about the severity of your symptoms<br>□ Believes service provider expertise<br>□ Others (specify) ________________	□<br>Refer for diagnosis<br>□ Refer for management<br>□ Repeat visit for treatment<br>□ No follow up planned/I refer myself for next visit
14	1. Pharmacy<br>2. Drug store3. Traditional/alternative/herbal medicine<br>4. General practitioner in private practice<br>5. Specialist in private practice<br>6. Public health center, write the name ___________<br>7. Clinic, write the name ___________<br>8. Private hospital, write the name _______________<br>9. BBKPM Bandung<br>10. Rotinsulu Pulmonary	1. Government<br>2. Private<br>3. Religious organization<br>4. NGOs/Ormas<br>5. Other____	□ None<br>□ Sputum examination □ X-ray/other imaging □ Blood test □ Other (specify) ______<br>□ Microscopic examination of sputum smear<br>□ Culture<br>□ Drug sensitivity test □ GeneXpert<br>□ Others (specify) __________________	□ None<br>□ Pain relief<br>□ Cough reliever<br>□ Antibiotics, specify _______<br>□ Corticosteroids<br>□ Herbal medicine<br>□ DM medicine<br>□ Can't remember the medicine<br>□ Other (specify) _______________	□ Referral by previous service provider<br>□ Free/affordable<br>□ Seek treatment for DM at this service provider<br>□ Have had treatment with this service provider (for reasons other than DM)<br>□ Distance/easy access<br>□ Recommended by relatives, friends, or colleagues<br>□ Medication available at these providers<br>□ Diagnostic tests are available at these providers<br>□ Diagnostic tests & medications are available at the same providers<br>□ Mild symptoms	□<br>Refer for diagnosis<br>□ Refer for management<br>□ Repeat visit for treatment<br>□ No follow up planned/I refer myself for next visit

No	Type of service provider (circle the appropriate one)	Hospital sector affiliation (circle the appropriate ONE)	Examinations examined at this service provider (check ALL that are appropriate, ask if the patient has a photo of the results of the examination)	Medicines given by this service provider (tick ALL that apply)	Reason for visit to this service provider (tick ALL that apply)	Follow-up plan by healthcare provider (check ALL that apply)
	Hospital<br>1 1. Regional general hospital, write the name ___________<br>1 2. RSUP Dr. Hasan Sadikin<br>1 3. Others (please specify)				□ Feels that the symptoms you are experiencing are related to witchcraft or the occult<br>□ Worried about the severity of your symptoms<br>□ Believes service provider expertise<br>□ Others (specify)	
15	1. Pharmacy<br>2. Drug store3. Traditional/alternative/herbal medicine<br>4. General practitioner in private practice<br>5. Specialist in private practice<br>6. Public health center, write the name ___________<br>7. Clinic, write the name ___________<br>8. Private hospital, write the name _______________<br>9. BBKPM Bandung<br>10. Rotinsulu Pulmonary Hospital<br>1 1. Regional general hospital, write the name ___________<br>1 2. RSUP Dr. Hasan Sadikin<br>1 3. Others (please specify)	1. Government<br>2. Private<br>3. Religious organization<br>4. NGOs/Orma s<br>Other______	□ None<br>□ Sputum examination □ X-ray/other imaging □ Blood test □ Other (specify) ______<br>□ Microscopic examination of sputum smear<br>□ Culture<br>□ Drug sensitivity test □ GeneXpert<br>□ Others (specify) __________________	□ None<br>□ Pain relief<br>□ Cough reliever<br>□ Antibiotics, specify _______<br>□ Corticosteroids<br>□ Herbal medicine<br>□ DM medicine<br>□ Can't remember the medicine<br>□ Other (specify) _______________	□ Referral by previous service provider<br>□ Free/affordable<br>□ Seek treatment for DM at this service provider<br>□ Have had treatment with this service provider (for reasons other than DM)<br>□ Distance/easy access<br>□ Recommended by relatives, friends, or colleagues<br>□ Medication available at these providers<br>□ Diagnostic tests are available at these providers<br>□ Diagnostic tests & medications are available at the same providers<br>□ Mild symptoms<br>□ Feels that the symptoms you are experiencing are related to witchcraft or the occult<br>□ Worried about the severity of your symptoms<br>□ Believes service provider expertise<br>□ Others (specify) ________________	□<br>Refer for diagnosis<br>□ Refer for management<br>□ Repeat visit for treatment<br>□ No follow up planned/I refer myself for next visit

No	Type of service provider (circle the appropriate one)	Hospital sector affiliation (circle the appropriate ONE)	Examinations examined at this service provider (check ALL that are appropriate, ask if the patient has a photo of the results of the examination)	Medicines given by this service provider (tick ALL that apply)	Reason for visit to this service provider (tick ALL that apply)	Follow-up plan by healthcare provider (check ALL that apply)
16	1. Pharmacy<br>2. Drug store3. Traditional/alternative/herbal medicine<br>4. General practitioner in private practice<br>5. Specialist in private practice<br>6. Public health center, write the name ___________<br>7. Clinic, write the name ___________<br>8. Private hospital, write the name _______________<br>9. BBKPM Bandung<br>10. Rotinsulu Pulmonary Hospital<br>1 1. Regional general hospital, write the name ___________<br>1 2. RSUP Dr. Hasan Sadikin<br>1 3. Others (please specify)	1. Government<br>2. Private<br>3. Religious organization<br>4. NGOs/Ormas<br>5. Other____	□ None<br>□ Sputum examination □ X-ray/other imaging □ Blood test □ Other (specify) ______<br>□ Microscopic examination of sputum smear<br>□ Culture<br>□ Drug sensitivity test □ GeneXpert<br>□ Others (specify) ___________________	□ None<br>□ Pain relief<br>□ Cough reliever<br>□ Antibiotics, specify _______<br>□ Corticosteroids<br>□ Herbal medicine<br>□ DM medicine<br>□ Can't remember the medicine<br>□ Other (specify) _______________	□ Referral by previous service provider<br>□ Free/affordable<br>□ Seek treatment for DM at this service provider<br>□ Have had treatment with this service provider (for reasons other than DM)<br>□ Distance/easy access<br>□ Recommended by relatives, friends, or colleagues<br>□ Medication available at these providers<br>□ Diagnostic tests are available at these providers<br>□ Diagnostic tests & medications are available at the same providers<br>□ Mild symptoms<br>□ Feels that the symptoms you are experiencing are related to witchcraft or the occult<br>□ Worried about the severity of your symptoms<br>□ Believes service provider expertise<br>□ Others (specify) _________________	□<br>Refer for diagnosis<br>□ Refer for management<br>□ Repeat visit for treatment<br>□ No follow up planned/I refer myself for next visit
17	1. Pharmacy<br>2. Drug store3. Traditional/alternative/herbal medicine<br>4. General practitioner in private practice<br>5. Specialist in private practice<br>6. Public health center, write the name ___________<br>7. Clinic, write the name ___________<br>8. Private hospital, write the name _______________<br>9. BBKPM Bandung	1. Government<br>2. Private<br>3. Religious organization<br>4. NGOs/Ormas<br>5. Other____	□ None<br>□ Sputum examination □ X-ray/other imaging □ Blood test □ Other (specify) ______<br>□ Microscopic examination of sputum smear<br>□ Culture<br>□ Drug sensitivity test □ GeneXpert<br>□ Others (specify) ___________________	□ None<br>□ Pain relief<br>□ Cough reliever<br>□ Antibiotics, specify _______<br>□ Corticosteroids<br>□ Herbal medicine<br>□ DM medicine<br>□ Can't remember the medicine<br>□ Other (specify) _______________	□ Referral by previous service provider<br>□ Free/affordable<br>□ Seek treatment for DM at this service provider<br>□ Have had treatment with this service provider (for reasons other than DM)<br>□ Distance/easy access<br>□ Recommended by relatives, friends, or colleagues<br>□ Medication available at these providers<br>□ Diagnostic tests are available at these providers<br>□ Diagnostic tests & medications are available at the same providers	□<br>Refer for diagnosis<br>□ Refer for management<br>□ Repeat visit for treatment<br>□ No follow up planned/I refer myself for next visit

No	Type of service provider (circle the appropriate one)	Hospital sector affiliation (circle the appropriate ONE)	Examinations examined at this service provider (check ALL that are appropriate, ask if the patient has a photo of the results of the examination)	Medicines given by this service provider (tick ALL that apply)	Reason for visit to this service provider (tick ALL that apply)	Follow-up plan by healthcare provider (check ALL that apply)
	10 . Rotinsulu Pulmonary Hospital<br>1 1. Regional general hospital, write the name ___________<br>1 2. Dr. Hasan Sadikin<br>1 3. Others (please specify)				□ Mild symptoms<br>□ Feels that the symptoms you are experiencing are related to witchcraft or the occult<br>□ Worried about the severity of your symptoms<br>□ Believes service provider expertise<br>□ Others (specify)	
18	1. Pharmacy<br>2. Drug store3. Traditional/alternative/herbal medicine<br>4. General practitioner in private practice<br>5. Specialist in private practice<br>6. Public health center, write the name ___________<br>7. Clinic, write the name ___________<br>8. Private hospital, write the name _______________<br>9. BBKPM Bandung<br>10. Rotinsulu Pulmonary Hospital<br>1 1. Regional general hospital, write the name ___________<br>1 2. RSUP Dr. Hasan Sadikin<br>1 3. Others (please specify)	1. Government<br>2. Private<br>3. Religious organization<br>4. NGOs/Ormas<br>5. Other____	□ None<br>□ Sputum examination □ X-ray/other imaging □ Blood test □ Other (specify) ______<br>□ Microscopic examination of sputum smear<br>□ Culture<br>□ Drug sensitivity test □ GeneXpert<br>□ Others (specify) __________________	□ None<br>□ Pain relief<br>□ Cough reliever<br>□ Antibiotics, specify _______<br>□ Corticosteroids<br>□ Herbal medicine<br>□ DM medicine<br>□ Can't remember the medicine<br>□ Other (specify) _______________	□ Referral by previous service provider<br>□ Free/affordable<br>□ Seek treatment for DM at this service provider<br>□ Have had treatment with this service provider (for reasons other than DM)<br>□ Distance/easy access<br>□ Recommended by relatives, friends, or colleagues<br>□ Medication available at these providers<br>□ Diagnostic tests are available at these providers<br>□ Diagnostic tests & medications are available at the same providers<br>□ Mild symptoms<br>□ Feels that the symptoms you are experiencing are related to witchcraft or the occult<br>□ Worried about the severity of your symptoms<br>□ Believes service provider expertise<br>□ Others (specify) ________________	□<br>Refer for diagnosis<br>□ Refer for management<br>□ Repeat visit for treatment<br>□ No follow up planned/I refer myself for next visit

No	Type of service provider (circle the appropriate one)	Hospital sector affiliation (circle the appropriate ONE)	Examinations examined at this service provider (check ALL that are appropriate, ask if the patient has a photo of the results of the examination)	Medicines given by this service provider (tick ALL that apply)	Reason for visit to this service provider (tick ALL that apply)	Follow-up plan by healthcare provider (check ALL that apply)
19	1. Pharmacy<br>2. Drug store3. Traditional/alternative/herbal medicine<br>4. General practitioner in private practice<br>5. Specialist in private practice<br>6. Public health center, write the name ___________<br>7. Clinic, write the name ___________<br>8. Private hospital, write the name _______________<br>9. BBKPM Bandung<br>10. Rotinsulu Pulmonary Hospital<br>1 1. Regional general hospital, write the name ___________<br>1 2. RSUP Dr. Hasan Sadikin<br>1 3. Others (please specify)	1. Government<br>2. Private<br>3. Religious organization<br>4. NGOs/Ormas<br>5. Other____	□ None<br>□ Sputum examination □ X-ray/other imaging □ Blood test □ Other (specify) ______<br>□ Microscopic examination of sputum smear<br>□ Culture<br>□ Drug sensitivity test □ GeneXpert<br>□ Others (specify) __________________	□ None<br>□ Pain relief<br>□ Cough reliever<br>□ Antibiotics, specify _______<br>□ Corticosteroids<br>□ Herbal medicine<br>□ DM medicine<br>□ Can't remember the medicine<br>□ Other (specify) _______________	□ Referral by previous service provider<br>□ Free/affordable<br>□ Seek treatment for DM at this service provider<br>□ Have had treatment with this service provider (for reasons other than DM)<br>□ Distance/easy access<br>□ Recommended by relatives, friends, or colleagues<br>□ Medication available at these providers<br>□ Diagnostic tests are available at these providers<br>□ Diagnostic tests & medications are available at the same providers<br>□ Mild symptoms<br>□ Feels that the symptoms you are experiencing are related to witchcraft or the occult<br>□ Worried about the severity of your symptoms<br>□ Believes service provider expertise<br>□ Others (specify) ________________	□<br>Refer for diagnosis<br>□ Refer for management<br>□ Repeat visit for treatment<br>□ No follow up planned/I refer myself for next visit
20	1. Pharmacy<br>2. Drug store3. Traditional/alternative/herbal medicine<br>4. General practitioner in private practice<br>5. Specialist in private practice<br>6. Public health center, write the name ___________<br>7. Clinic, write the name ___________<br>8. Private hospital, write the name _______________<br>9. BBKPM Bandung<br>10. Rotinsulu Pulmonary	1. Government<br>2. Private<br>3. Religious organization<br>4. NGOs/Ormas<br>5. Other____	□ None<br>□ Sputum examination □ X-ray/other imaging □ Blood test □ Other (specify) ______<br>□ Microscopic examination of sputum smear<br>□ Culture<br>□ Drug sensitivity test □ GeneXpert<br>□ Others (specify) __________________	□ None<br>□ Pain relief<br>□ Cough reliever<br>□ Antibiotics, specify _______<br>□ Corticosteroids<br>□ Herbal medicine<br>□ DM medicine<br>□ Can't remember the medicine<br>□ Other (specify) _______________	□ Referral by previous service provider<br>□ Free/affordable<br>□ Seek treatment for DM at this service provider<br>□ Have had treatment with this service provider (for reasons other than DM)<br>□ Distance/easy access<br>□ Recommended by relatives, friends, or colleagues<br>□ Medication available at these providers<br>□ Diagnostic tests are available at these providers<br>□ Diagnostic tests & medications are available at the same providers<br>□ Mild symptoms	□<br>Refer for diagnosis<br>□ Refer for management<br>□ Repeat visit for treatment<br>□ No follow up planned/I refer myself for next visit

No	Type of service provider (circle the appropriate one)	Hospital sector affiliation (circle the appropriate ONE)	Examinations examined at this service provider (check ALL that are appropriate, ask if the patient has a photo of the results of the examination)	Medicines given by this service provider (tick ALL that apply)	Reason for visit to this service provider (tick ALL that apply)	Follow-up plan by healthcare provider (check ALL that apply)
	Hospital<br>1 1. Regional general hospital, write the name ___________<br>1 2. RSUP Dr. Hasan Sadikin<br>1 3. Others (please specify)				☐ Feels that the symptoms you are experiencing are related to witchcraft or the occult<br>☐ Worried about the severity of your symptoms<br>☐ Believes service provider expertise<br>☐ Others (specify)	

Part III. Time and cost to DR-TB diagnosis

Patient time and cost

27. How much time and money did you spend at each healthcare provider?

Fill in one line per healthcare provider. Fill in the costs in rupiah. **Please circle the health care provider where you were diagnosed with DR-TB.**
Please only write down the expenses you incur for yourself. Expenses for companions will be asked in the next section.

No	Number of visits to this service provider	Time spent (On average)		Total personal expenses (overall) for each health care provider in Rupiah (fill in expenses incurred for you only)										
				Administrative costs (registration, consultation)	Chest X- ray and other imaging	Lab examination (Other than Chest X-ray)	Medication (TB-related symptoms)	Supplementary drugs (i.e. nutritional vitamin)	Hospitalization (all costs if any inpatient care occurred)	Other medical procedures (specify)	Travel (Return total per visit, including parking fees)	Food or Meals (total per visit)	Accomodation	Other expenses (i.e. linen, soap, other services)
1	______time	_____ hours _____ minutes	______hours _____ minutes If treated: ______days							Type: _____ Cost: Rp_____				Type: _____ Cost: Rp_____
2	______time	_____ hours _____ minutes	______hours _____ minutes If treated: ______days							Type: _____ Cost: Rp_____				Type: _____ Cost: Rp_____
3	______time	_____ hours _____ minutes	______hours _____ minutes If treated: ______days							Type: _____ Cost: Rp_____				Type: _____ Cost: Rp_____

4	______time	_____<br>hours<br>_____<br>minutes	______hours<br>_____<br>minutes<br>If treated:<br>______days							Type: _____<br>Cost:<br>Rp____				Type: _____<br>Cost:<br>Rp____
6	______time	_____<br>hours<br>_____<br>minutes	______hours<br>_____<br>minutes<br>If treated:<br>______days							Type: _____<br>\<br>Cost:<br>Rp____				Type: _____<br>Cost:<br>Rp____
7	______time	_____<br>hours<br>_____<br>minutes	______hours<br>_____<br>minutes<br>If treated:<br>______days							Type: _____<br>\<br>Cost:<br>Rp____				Type: _____<br>Cost:<br>Rp____
8	______time	_____<br>hours<br>_____<br>minutes	______hours<br>_____<br>minutes<br>If treated:<br>______days							Type: _____<br>\<br>Cost:<br>Rp____				Type: _____<br>Cost:<br>Rp____
9	______time	_____<br>hours<br>_____<br>minutes	______hours<br>_____<br>minutes							Type: _____<br>\<br>Cost:				Type: _____<br>Cost:<br>Rp____

										Rp____				
			If treated:<br>______days											
10	______time	_____<br>hours<br>____<br>minutes	______hours<br>____<br>minutes<br>If treated:<br>______days							Type: _____<br>\<br>Cost:<br>Rp____				Type: _____<br>Cost:<br>Rp____
11	______time	_____<br>hours<br>____<br>minutes	______hours<br>____<br>minutes<br>If treated:<br>______days							Type: _____<br>\<br>Cost:<br>Rp____				Type: _____<br>Cost:<br>Rp____
12	______time	_____<br>hours<br>____<br>minutes	______hours<br>____<br>minutes<br>If treated:<br>______days							Type: _____<br>\<br>Cost:<br>Rp____				Type: _____<br>Cost:<br>Rp____
13	______time	_____<br>hours<br>____<br>minutes	______hours<br>____<br>minutes<br>If treated:<br>______days							Type: _____<br>\<br>Cost:<br>Rp____				Type: _____<br>Cost:<br>Rp____

14	______time	_____<br>hours<br>_____<br>minutes	______hours<br>____<br>minutes<br>If treated:<br>______days							Type: _____<br>\<br>Cost:<br>Rp____				Type: _____<br>Cost:<br>Rp____

15	______time	_____<br>hours<br>_____<br>minutes	______hours<br>____<br>minutes<br>If treated:<br>______days							Type: _____<br>\<br>Cost:<br>Rp____				Type: _____<br>Cost:<br>Rp_____
16	______time	_____<br>hours<br>_____<br>minutes	______hours<br>____<br>minutes<br>If treated:<br>______days							Type: _____<br>\<br>Cost:<br>Rp____				Type: _____<br>Cost:<br>Rp_____
17	______time	_____<br>hours<br>_____<br>minutes	______hours<br>____<br>minutes<br>If treated:<br>days							Type: _____<br>\<br>Cost:<br>Rp____				Type: _____<br>Cost:<br>Rp_____

18	______time	_____ hours ____ minutes	______hours ____ minutes If treated: days							Type: _____ \ Cost: Rp____				Type: _____ Cost: Rp_____
19	______time	_____ hours ____ minutes	______hours ____ minutes If treated: ______days							Type: _____ \ Cost: Rp____				Type: _____ Cost: Rp_____

Companion & Visitor Fees

Before the diagnosis of DR-TB

**28. Are you accompanied by friends or relatives on visits to health care providers for TB symptoms?**	☐ Yes ☐ No *(Go to P29)*
**b) If yes, on how many visits did this person accompany you?**	__________visits
**c) Does your driver live in the same house as you?**	☐ Yes ☐ No

d) On average, how much <u>additional cost per visit</u> was incurred for each introduction (friend or relatives)?

Travel (round trip, only if there is an additional fee you pay)	Rp ________________
Accommodation	Rp ________________
Food or Meals	Rp ________________
Other expenses (specify) ________________	Rp ________________

**29. If you were hospitalized <u>before your DR-TB diagnosis</u>, were you accompanied by friends or relatives at the hospital** (who slept with you at the hospital)**?**	☐ Yes<br>☐ No *(Go to P30)*<br>☐ Not hospitalized *(Go to P31)*
**b) If yes, how many days did your companion stay with you at the hospital** (sleep there)**?**	_________day
**c) Does your companion live in the same house as you?**	☐ Yes ☐ No

d) On average, how much extra do you incur for each companion (friend or relative) staying with you in the hospital?

Travel (during inpatient care, including parking fee)	Rp ___________
Accommodation	Rp___________
Food or meals (during inpatient care)	Rp____________________
Other expenses (specify) ___________	Rp____________________

**30. Did any of your friends or relatives <u>visited</u> you in the hospital?**	☐ Yes<br>☐ No *(Go to P31)*<br>☐ No hospitalized *(Go to P31)*
**b) If yes, how many times have you been visited by friends or other relatives? (total)**	_________time
**c) Do most of your visitors live in the same house as you?**	☐ Yes ☐ No
**d) What is the average time required for each of these visits (including travel time)?**	__________hours _________minutes

e) What is the average cost per visit of your friends or relatives?

Transportation (round trip, including parking fee)	Rp _______________
Accommodation	Rp _______________
Food or Meals (total per visit)	Rp _______________

Other xpenses (specify) ______________ Rp _______________		
Visits for DR-TB diagnosis		
**31. Were you accompanied by friends or relatives on visits for DR-TB diagnosis?**	☐ Yes	☐ No *(Go to P32)*
**b) Does your driver live in the same house as you?**	☐ Yes	☐ No
**c) What <u>is the additional cost</u> incurred for each companion (friend or relative) on this visit?**<br><br>Transportation (round trip, only if there is an additional fee you pay) Rp _________________<br>Accommodation (hospital or other) Rp _________________<br>Food or meals (total per visit) Rp _________________<br>Other expenses (specify) ________________ Rp _________________		

Reimbursement	
**32. Do you receive *reimbursement* for expenses incurred <u>prior to DR-TB diagnosis</u>?**	☐ Yes ☐ No *(Go to P33)*
**b) If yes, what was the source of the reimbursement and how much was the total reimbursement/assistance you received before being diagnosed with DR-TB?** (check all that apply and state the total amount of each reimbursement)<br><br>☐ STPI (*enabler* from Terjang) Rp ___________________<br>☐ BPJS Health/Government Insurance Rp ___________________<br>☐ Private health insurance Rp ___________________<br>☐ Corporate health insurance Rp _______<br>☐ Others (please specify) ______________ Rp _______	
**33. Do you receive reimbursement for the costs you incur <u>for DR-TB diagnosis</u>?**	☐ Yes ☐ No
**b) If yes, what is the source of the reimbursement and how much total reimbursement/assistance did you get before DR-TB diagnosis?** (check all that apply and state the total amount of each reimbursement)<br><br>☐ STPI (*enabler* from Terjang) Rp ___________________<br>☐ BPJS Health/Government Insurance Rp ___________________<br>☐ Private health insurance Rp ___________________<br>☐ Corporate health insurance Rp __________________<br>☐ Others (please specify) ______________ Rp ________________	

Part III. Socioeconomic Information

Occupation and Income	
**34. Who is the primary income earner in the household?**	1. Patient<br>2. Others (specify) ___________
**35. What was your main occupation <u>before DR-TB diagnosis</u>?**	1. Formal sector jobs<br>2. Informal sector jobs<br>3. No job yet/looking for work<br>4. Retired<br>5. Housewife<br>6. Student / student<br>7. Other (specify) ______________________
**b) For how long did you work <u>before DR-TB diagnosis</u>?**	____________hours per week
**c) On average, how much was your personal take home earnings per month <u>before DR-TB diagnosis</u>?**	Rp ______________per month
**36. Are there any changes in your occupation <u>after you were diagnosed with DR-TB</u>?**	☐ Yes, I change my job<br>☐ Yes, I stop working<br>☐ No change
**37. What is your main occupation <u>now</u>?**	1. Formal sector jobs<br>2. Informal sector jobs<br>3. No job yet/looking for work<br>4. Retired<br>5. Housewife<br>6. Student / student<br>7. Other (specify) ____

**b) For how long do you work <u>now</u>?**	____________hours per week
**c) On average, how much is your personal take home earnings per month <u>now</u>?**	Rp __________________per month
**38. What was your household's average monthly income <u>before you were diagnosed with DR-TB</u>?** *(Please include the income of all household members, except yourself)*	Rp _________________per month
**39. What was your household's average monthly income <u>now</u>?** *(Please include the income of all household members, except yourself)*	Rp _________________per month
**b) If the income on question 38 differs from question 39, is this changed due to DR-TB illnesses?**	☐ Yes<br>☐ No
**40. Do any of your children of or below school age, work or are not able to attend school due to your DR-TB illness?**	☐ Yes<br>☐ No

Coping Mechanisms	
**41. Did you borrow any money to cover costs due to your DR-TB illness?** (from relatives, friends, bank, etc)	☐ Yes ☐ No *(Go to P42)*
**b) If yes, how much did you borrow?**	Rp _______________
**c) From whom did you borrow this money?**	1. Family<br>2. Neighbours/Friends<br>3. Bank Loan<br>4. Cooperatives<br>5. Others (Specify) ________
**c) What is the amount of interest on debt? (%)**	1. … %<br>2. I did not pay for interest<br>3. I was not asked to repay the loan
**42. Have you sold any of your property to finance the cost of your DR-TB illness?** *(i.e. jewelry, vehicle, and other household items)*	☐ Yes ☐ No
**b) How much did you earn from the sale of all above items mentioned?**	Rp _______________
